# Supplementary material for: Role of Ribosomal Protein bS1 in Orthogonal mRNA Start Codon Selection
Source: Biochemistry. 2025 Jan 24;64(3):710–8. doi: 10.1021/acs.biochem.4c00688 (PMC11800381; doi:10.1021/acs.biochem.4c00688)
Supplement: Supplementary file 1 — bi4c00688_si_001.pdf [file bi4c00688_si_001.pdf]

Supporting Information for

Role of ribosomal protein bS1 in orthogonal mRNA start codon selection

Kristina V. Boyko[1], Rebecca A. Bernstein[2], Minji Kim[3], Jamie H. D. Cate[2,3,4,5,*]

[1]Biophysics Graduate Group, University of California, Berkeley, CA 94720, USA

[2]Department of Chemistry, University of California, Berkeley, CA 94720, USA

[3]Department of Molecular and Cell Biology, University of California, Berkeley, CA 94720, USA

[4]Innovative Genomics Institute, University of California, Berkeley, CA 94720, USA

[5]Molecular Biophysics and Integrated Bioimaging, Lawrence Berkeley National Laboratory, Berkeley, CA 94720, USA

[*]Corresponding author. Email: j-h-doudna-cate@berkeley.edu

Table of Contents

1. Supplementary tables
    a. Table S1: Cryo-EM statistics
    b. Table S2: Luminescence data
    c. Table S3: Orthogonality luminescence data

2. Supplementary figures
    a. Figure S1. RT-PCR of ribosome mutants
    b. Figure S2. Cryo-EM processing workflow.
    c. Figure S3. Fourier shell correlation (FSC) curves of the S1V3 map.
    d. Figure S4. Reassociation of S1V4 30S subunits with wild-type 50S subunits.
    e. Figure S5. Fourier shell correlation (FSC) curves of the S1V4 map.
    f. Figure S6. Local resolution of the S1V4 30S subunit cryo-EM map.
    g. Figure S7. RT-PCR of mutant ribosomes.
    h. Figure S8. *In vivo* activity of S1V4 30S subunits.

SUPPLEMENTARY TABLES

	S1V3	S1V4
Model resolution (Å)	2.56	1.80
FSC threshold	0.5	0.5
Total non-hydrogen atoms	51540	51586
R.m.s. deviations from ideal values		
Bond (Å)	0.005	0.009
Angle (°)	0.816	1.22
Molprobity score	2.43	2.42
Clash score	13	13
Romater outliers (%)	3.56	3.66
Ramachandran plot		
Favored (%)	94.92	94.92
Allowed (%)	5.03	5.03
Outliers (%)	0.04	0.04
RNA validation		
Angles outliers (%)	0.043	0.061
Sugar pucker outliers (%)	0.265	0.027
Average suiteness	0.541	0.536

Supplementary Table S1. Cryo-EM statistics. Overall map and model statistics for the S1V3 and S1V4 structures.

Raw luminescence reading

	WT 30S	S1V1	50S control		WT 30S	S1V2	50S control
Trial 1	376746	45038	9041	Trial 1	53712	14293	1777
Trial 2	895706	191713	268	Trial 2	68803	17277	1787
Trial 3	628275	164591	234	Trial 3	73249	17878	1950
	WT 30S	S1V3	50S control		WT 30S	S1V4	50S control
Trial 1	1838	514	15	Trial 1	1838	857	15
Trial 2	129670	42865	28	Trial 2	129670	69507	28
Trial 3	125774	53260	29	Trial 3	125774	64947	29

Percent activity normalized to WT 30S

	WT 30S	S1V1	50S control		WT 30S	S1V2	50S control
Trial 1	100.00	11.95	0.02	Trial 1	100.00	26.61	0.03
Trial 2	100.00	21.40	0.00	Trial 2	100.00	25.11	0.03
Trial 3	100.00	26.20	0.00	Trial 3	100.00	24.41	0.03
	WT 30S	S1V2	50S control		WT 30S	S1V4	50S control
Trial 1	100.00	27.99	0.82	Trial 1	100.00	46.64	0.82
Trial 2	100.00	33.06	0.02	Trial 2	100.00	53.60	0.02
Trial 3	100.00	42.35	0.02	Trial 3	100.00	51.64	0.02

Supplementary Table S2. Luminescence data. Raw and normalized data of *in vitro* translation reactions. Trials 2 and 3 were carried out for S1V3 and S1V4 with the same WT 30S control.

Raw luminescence reading

	WT 30S (ASD + SD)	WT 30S (ASD + oSD)	o30S (oASD + SD)	o30S (oASD + oSD)
Trial 1	6991401	4201	26350	2339187
Trial 2	8496880	3666	26894	2138661
Trial 3	9108992	4089	31457	2197034
	S1V4 (ASD + SD)	S1V4 (ASD + oSD)	oS1V4 (oASD + SD)	oS1V4 (oASD + oSD)
Trial 1	2339187	294691	19444	1785091
Trial 2	2138661	284424	16627	1727275
Trial 3	2197034	328052	16215	1856041

Percent activity normalized to WT 30S

	WT 30S (ASD + SD)	WT 30S (ASD + oSD)	o30S (oASD + SD)	o30S (oASD + oSD)
Trial 1	85.00	0.06	0.38	49.36
Trial 2	103.00	0.05	0.38	47.65
Trial 3	118.00	0.06	0.45	47.35
	S1V4 (ASD + SD)	S1V4 (ASD + oSD)	oS1V4 (oASD + SD)	oS1V4 (oASD + oSD)
Trial 1	33.46	4.22	0.28	25.53
Trial 2	30.59	4.07	0.24	24.71
Trial 3	31.43	4.69	0.23	26.55

Supplementary Table S3. Orthogonality luminescence data. Raw and normalized data for *in vitro* orthogonality experiments.

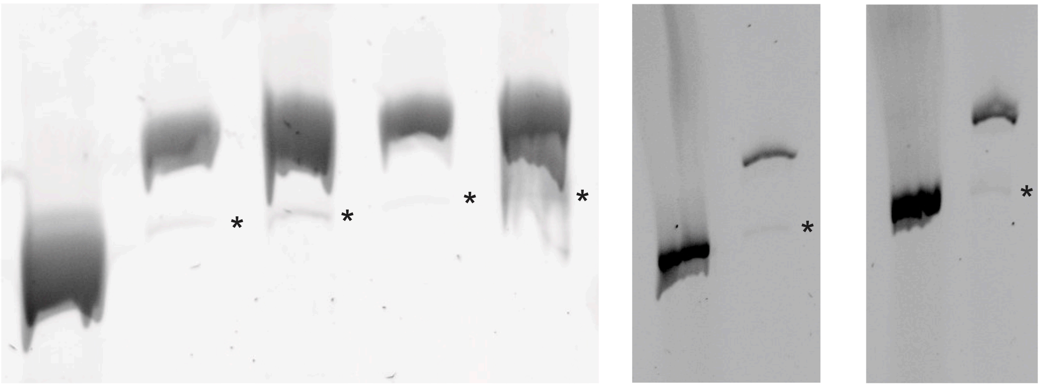

Supplementary Figure S1. RT-PCR of ribosome mutants. 10% TBE gel of cDNA from RT-PCR flanking the MS2 tag in the 16S rRNA. The lower band indicates wild-type 16S contamination, marked by asterisks. In all mutants, the tagged ribosomes have less than 3% wild-type contamination.

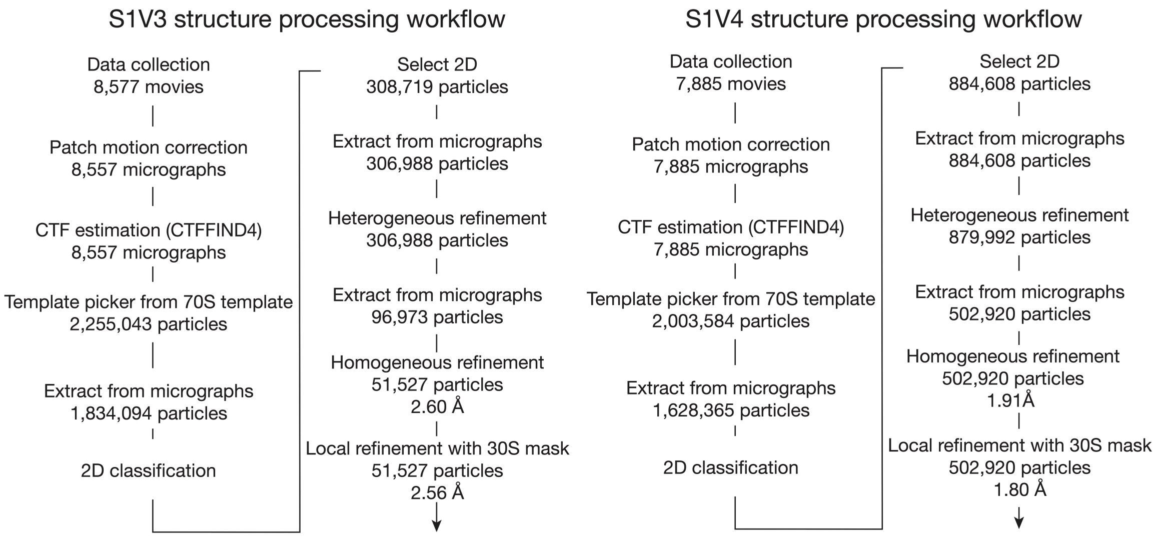

Supplementary Figure S2. Cryo-EM processing workflow. Cryo-EM data processing for both the S1V4 and S1V3 maps were done in CryoSPARC 4.[26] The number of movies, micrographs, or particles is listed at each step in the workflow. Resolutions are reported with an FSC threshold of 0.143.

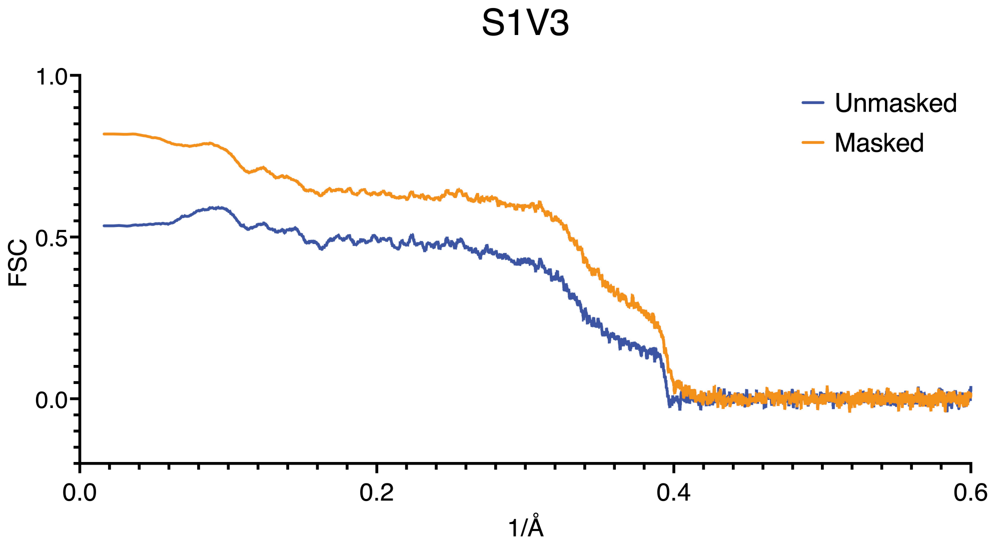

Supplementary Figure S3. Fourier shell correlation (FSC) curves of the S1V3 map. The FSC curve for the masked map (30S subunit only) is indicated in orange and the unmasked map (70S ribosome) is indicated in blue.

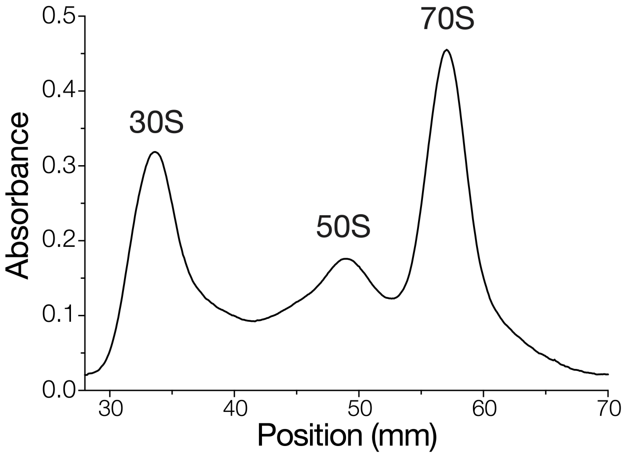

Supplementary Figure S4. Reassociation of S1V4 30S subunits with wild-type 50S subunits. 20-40% reassociation gradient of 1000 nM S1V4 30S subunits with 500 nM wild-type 50S subunits in 10 mM $MgCl_2$ and used for subsequent cryo-EM studies.

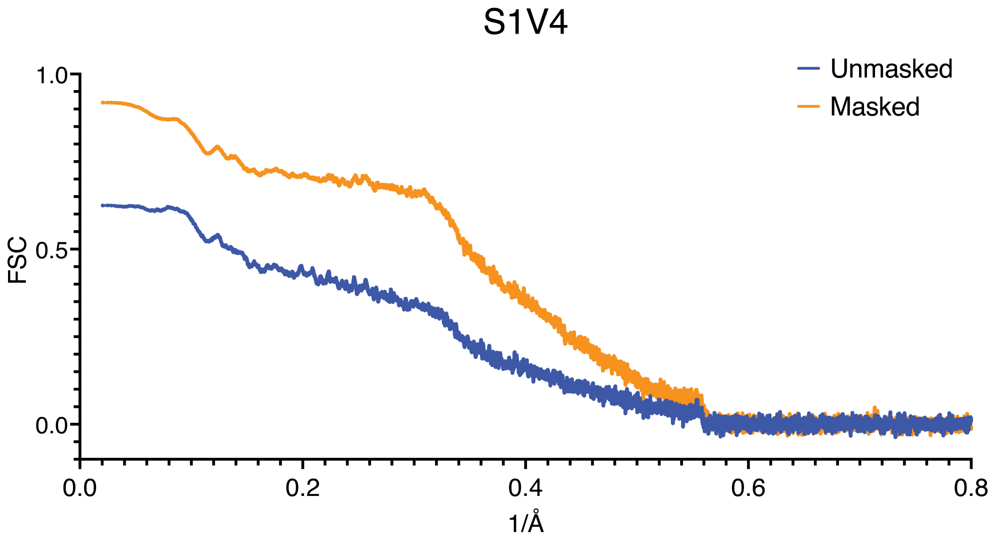

Supplementary Figure S5. Fourier shell correlation (FSC) curves of the S1V4 map. The FSC curves for the masked map (30S subunit only) is indicated in orange and the unmasked map (70S ribosome) is indicated in blue.

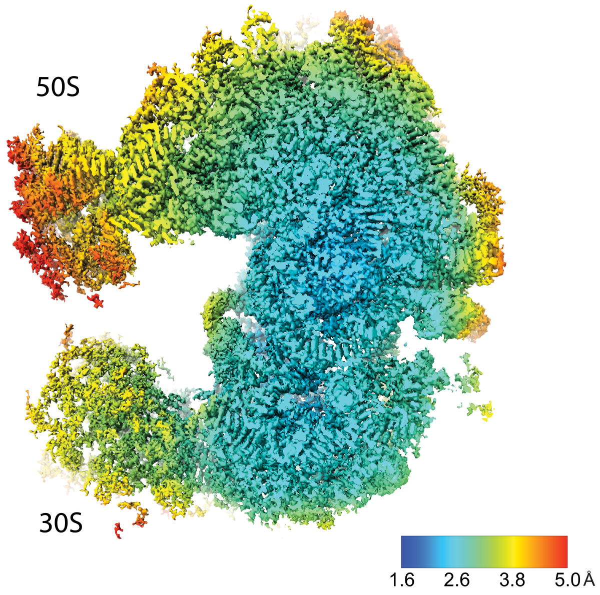

Supplementary Figure S6. Local resolution of the S1V4 30S subunit cryo-EM map. A cross-section of the local resolution was made in ChimeraX and the color key is reported in Ångstroms. Local resolution was measured using the Local Resolution estimation in Relion 4.0-beta-2.[29]

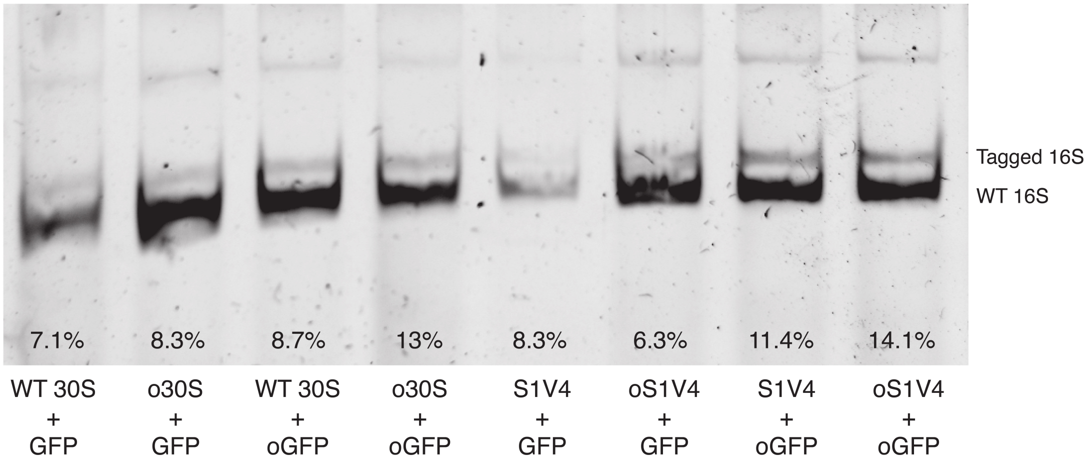

Supplementary Figure S7. RT-PCR of mutant ribosomes. 5% TBE gel of RT-PCR of mutant ribosomes used in *in vivo* experiments. RNA was extracted from cells four hours after induction with IPTG and arabinose. The lower band indicates wild-type 16S and the higher band is from the tagged 16S. The expected difference is 35 base pairs. Expression was quantified by integrating the gel bands, excluding the edges.

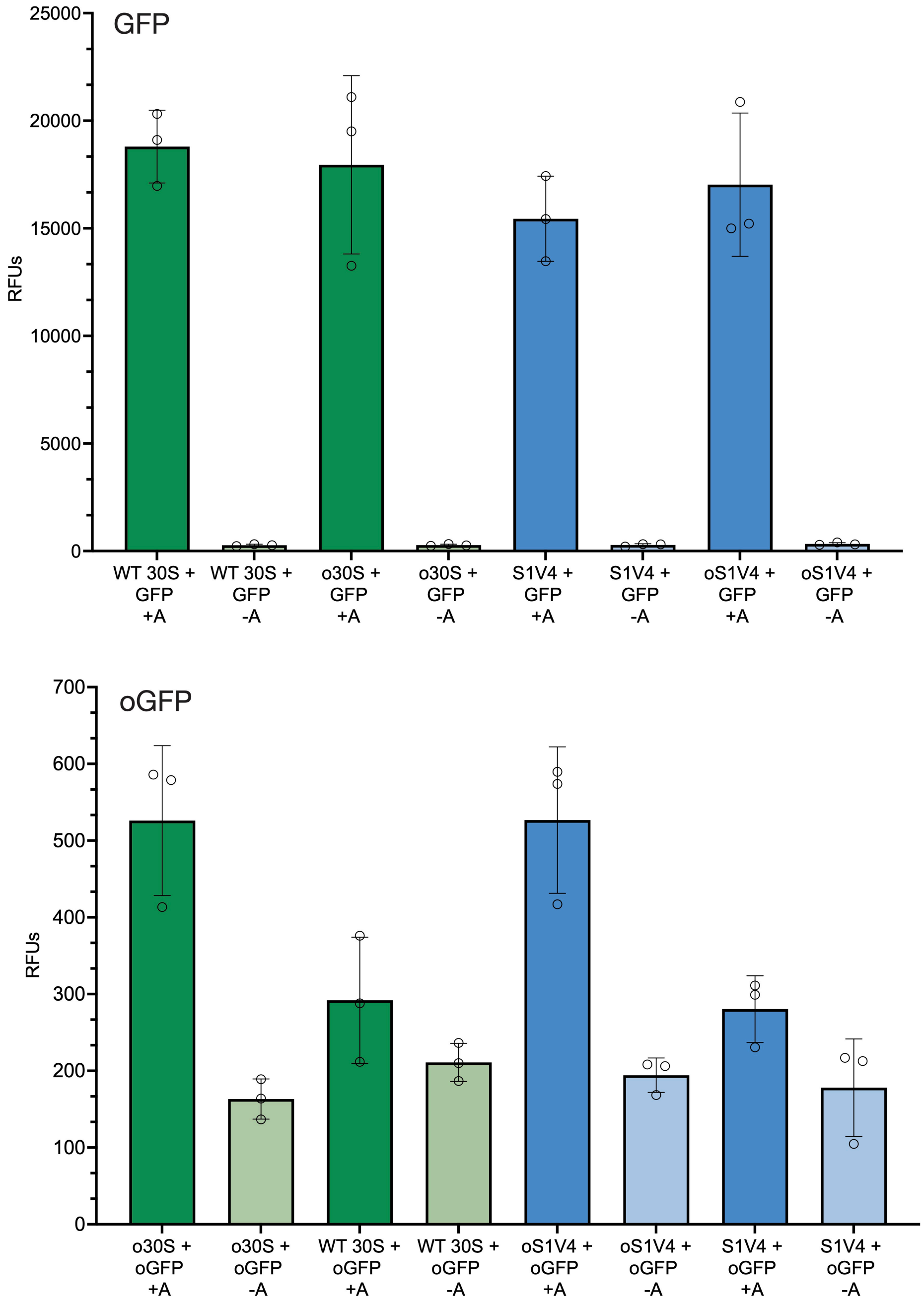

Supplementary Figure 8. *In vivo* activity of S1V4 30S subunits. Columns with the addition of arabinose (+A) and with no arabinose added (-A) are shown.
